# Validating an infrared thermal switch as a novel access technology

**DOI:** 10.1186/1475-925X-9-38

**Published:** 2010-08-05

**Authors:** Negar Memarian, Anastasios N Venetsanopoulos, Tom Chau

**Affiliations:** 1 Institute of Biomaterials and Biomedical Engineering, University of Toronto, Toronto, Canada; 2 Bloorview Research Institute, Holland-Bloorview Kids Rehabilitation Hospital, Toronto, Canada; 3 Department of Electrical and Computer Engineering, University of Toronto, Toronto, Canada; 4 Department of Electrical and Computer Engineering, Ryerson University, Toronto, Canada

## Abstract

**Background:**

Recently, a novel single-switch access technology based on infrared thermography was proposed. The technology exploits the temperature differences between the inside and surrounding areas of the mouth as a switch trigger, thereby allowing voluntary switch activation upon mouth opening. However, for this technology to be clinically viable, it must be validated against a gold standard switch, such as a chin switch, that taps into the same voluntary motion.

**Methods:**

In this study, we report an experiment designed to gauge the concurrent validity of the infrared thermal switch. Ten able-bodied adults participated in a series of 3 test sessions where they simultaneously used both an infrared thermal and conventional chin switch to perform multiple trials of a number identification task with visual, auditory and audiovisual stimuli. Participants also provided qualitative feedback about switch use. User performance with the two switches was quantified using an efficiency measure based on mutual information.

**Results:**

User performance (p = 0.16) and response time (p = 0.25) with the infrared thermal switch were comparable to those of the gold standard. Users reported preference for the infrared thermal switch given its non-contact nature and robustness to changes in user posture.

**Conclusions:**

Thermal infrared access technology appears to be a valid single switch alternative for individuals with disabilities who retain voluntary mouth opening and closing.

## Background

An access technology is a system that senses a physical movement or physiological change from a person and uses this information to drive a user interface [1]. This technology is particularly useful for individuals with severe motor impairments who lack a conventional means of access and communication. Examples of access technologies developed for this population are sip and puff switches [2], chin switches [3], computer vision-based systems [4,5], or electromyography-based systems [6,7]. While attempts to design and develop new access technologies are ongoing [8-11] an important challenge is to ensure that the new access technology is valid. Validity in the present context refers to the correlation between the switch activations of the access technology of interest and those of an established gold standard. This concept is similar to criterion validity [12]. In engineering design, validity testing is usually performed with prototypes to verify system functionality [13]. If an access technology fails to be valid, the user will find it frustrating to use, and hence the technology will quickly lose its appeal as an assistive device. On the other hand, if the system proves to be adequately valid, then poor performance of the access technology is most likely due to user error rather than technology error. In other words, validity testing is crucial to distinguish between user-dependent errors and algorithm-dependent errors.

In the present paper we describe and discuss empirical validity testing of an enhanced version of the infrared thermography based access technology proposed in [10]. We report an experiment designed to gauge the validity of this access technology with respect to a conventional chin switch. The chin switch was chosen as the benchmark for two reasons. Firstly, the chin switch is guaranteed to activate upon the application of force, and secondly, the chin switch can be activated by exactly the same motion that triggers the infrared thermal switch, i.e., mouth opening.

In the following sections, first, we explain the infrared thermal switch and its underlying computational algorithm. Next, we detail the experimental setup for the validation study and the ensuing data analysis. The paper will be rounded out with a presentation and discussion of the empirical results.

## Methods

### A. Infrared Thermal Switch

The infrared thermography-based access technology used in this study is an enhanced version of the system proposed in [10]. In particular, we employ a different motion estimation method and introduce a new anthropometric filter of non-mouth objects to reduce false positive activations. Also, we invoke additional software and hardware to realize a real time infrared thermographic single switch. This system captures the facial temperature distribution of a user with an infrared thermal camera and translates the user's voluntary mouth opening activity into a switch activation using a computerized algorithm.

#### A.1. Instrumentation

**Figure 1:**
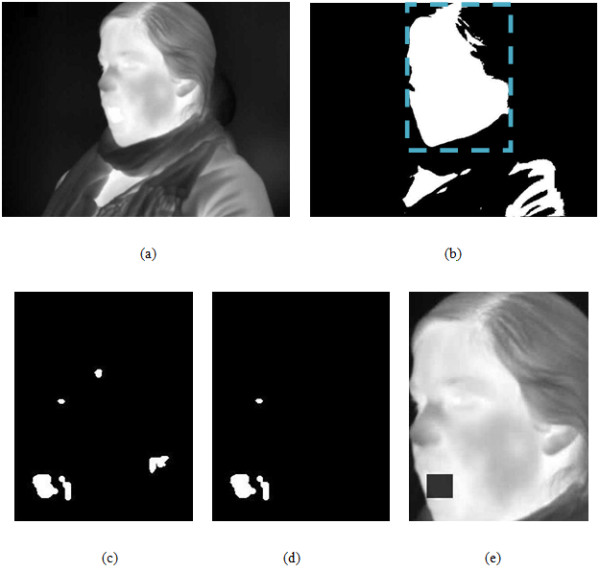
**Visual representation of the infrared thermal switch video processing algorithm**. (a) Input grayscale thermal video frame, (b) Result of face localization, (c) Result of intensity analysis (warm area mask), (d) Intersection of warm area mask and motion mask, (e) Open mouth detected and marked on the video. Note that for ease of visualization, images (c)-(e) only show the smaller region demarcated by the dashed box in (b).

The infrared thermal video was acquired in real time with a ThermaCAM SC640 by FLIR [14]. The camera had a thermal sensitivity of ≤60 mK and a resolution of 640 × 480 pixels. The acquired video was non-radiometric and grey scale, with bright intensities corresponding to warm regions and dark intensities corresponding to cold regions (Figure 1(a)). The video was sent to a DELL Inspiron 1560 laptop (Intel^® ^Core™2 CPU T7200 @ 2.00 GHz and 2.00 GB of RAM) via a fire wire cable for real time processing. Upon detection of a mouth open-close sequence by the video processing algorithm, a mouse click or key press was generated using a latching relay by DLP Design Inc. and a Swifty USB switch interface by Origin Instruments™.

#### A.2. Video Processing Algorithm

The video processing algorithm for the infrared thermal switch was implemented in SIMULINK R2008a. The algorithm consisted of three main modules, namely user face localization, motion and intensity analyses, and filtering of non-mouth objects. In the following, we describe an enhanced and online version of the original algorithm proposed in [10], with a new motion estimator and new anthropometric filters. We remind the reader that although enhancements to the original algorithm are presented below, the main focus of this study was the concurrent validity testing of the thermal switch, which is detailed in the subsequent section.

##### Face Localization

Given that the face is generally warmer than the surrounding environment, the face localization module began with an adaptive thresholding of the frame's grey scale intensities based on Otsu's method [15]. Thresholding was followed by the selection of the user's face, i.e., the largest round or semi-round object within the black and white thresholded image. Zero padding was used at all four sides of the frame to account for user head motion which may have caused the segmented facial region to overlap with the frame borders. Figure 1(a) and 1(b) show the original input video frame and the localized face within that frame, respectively.

##### Motion and Intensity Analyses

This phase of the algorithm is based on two key observations. First, the inside of the human mouth is typically warmer than the surrounding areas of the face. Consequently, an open mouth appears as a bright patch on the thermal video. Second, opening and closing of the mouth involves motion.

In order to detect the warm regions within the face, a second Otsu thresholding [15] was performed on the segmented face, this time with double the previous threshold value. This thresholding yielded the warm region mask, as shown in Figure 1(c).

For motion tracking, the sum of absolute differences (SAD) was used. By performing a two-dimensional SAD between the current and previous frames, we basically looked for similarity between the two consecutive images. Denote the current video frame as matrix *I *with dimensions (*M*_*i*_, *N*_*i*_) and the previous video frame to be matrix *T *with dimensions (*M*_*t*_, *N*_*t*_). Then the two-dimensional SAD matrix [16,17] is given by:

(1) $$SAD(j,k)=\sum_{m=0}^{\left(M_{t}-1\right)}\sum_{n=0}^{\left(N_{t}-1\right)}abs(I(m+j,n+k)-T(m,n))$$

Where

$$0\leq j<M_{i}-M_{t}+1$$

and

$$0\leq k<N_{i}-N_{t}+1$$

The greater the similarity between the two matrices, the smaller the SAD values. In contrast, motion results in dissimilarity and hence a bigger SAD value. The algorithm retained only those pixels in the frame whose SAD values fell in the motion range of mouth open/close activity. Each person opens his or her mouth at a different speed and so individualized SAD thresholds were selected during an initial calibration period. The motion analysis phase yielded a motion mask, which included all the pixels that have comparable motion to mouth opening and closing activity. Of course, this mask did not only include the mouth pixels, but also other areas that have similar motion (e.g., eyebrows, eye lids), hence, the need for a simultaneous warm, region mask. Figure 1(d) depicts the warm facial regions that also exhibited motion.

##### Filtering non-mouth objects

Motion and intensity analyses identified multiple objects as open mouth candidates. However not all of those objects represent a true open mouth. For example, the regions around the eyes, chin and forehead were commonly singled out. These objects passed the preceding intensity and motion filters because they were both warm [18] and moving. Thus in this last stage of the algorithm, non-mouth objects were filtered based on size, morphological, and anthropometric features. Specifically, if the object departed from one or more of the following conditions, it was flagged as a false positive by the system and subsequently removed.

1. 200 pixels <Area <1500 pixels

2. Eccentricity ≤0.9

3. (Area of object)/(Area of bounding box) >0.4

4. $\frac{1}{2}$ (menton sellion length) <Object's centroid <$\frac{3}{4}$ (menton sellion length)

5. $\frac{1}{3}$ (head breadth) <Object's centroid <$\frac{2}{3}$ (head breadth)

The first condition is basically a size filter that eliminated objects that were too small or too large to be an open mouth. The minimum and maximum size limits in this equation are an order of magnitude larger than the values previously reported in [10], because in the current study we use a higher resolution infrared thermal camera, and also place the camera closer to the participant to minimize background artifacts. The second and third conditions are morphological filters, which removed respectively, regions that were too long to qualify as a mouth and areas that were too hollow as the mouth was expected to be solid. The fifth condition is a newly introduced anthropometric filter. Based on human face anthropometry, the mouth is located in the lower half of the menton-sellion length and in the middle third of the head breadth [19]. The menton sellion length is the distance in the midsagittal plane between the menton landmark at the bottom of the chin and the sellion landmark at the deepest point of the nasal root depression. The head breadth is the maximum horizontal breadth of the head above the level of the ears [19,20].

Objects that did not satisfy the expected size, shape, and location of an open mouth within the face were filtered out in this phase. Finally the only object that satisfied all the above conditions was considered to be an open mouth. The bounding box of this object is marked on the video frame as shown in Figure 1(e).

### B. Validity Testing Experiment

#### B.1. Objective

The objective of this experiment was to validate the detection algorithm of the infrared thermal switch introduced above. The accuracy of the infrared thermal switch may be impacted by user error or algorithm error. An error is either a missed activation or a false alarm. For example a user-dependent miss is when the user does not open his/her mouth, when he/she is cued to do so, while an algorithm-dependent miss is when the user does voluntarily open his/her mouth upon cue, but the system does not detect it and thus does not activate the switch. A false activation can similarly be generated either because of the user opening his/her mouth at the wrong time or the algorithm erroneously picking up non-mouth objects as mouth opening. The infrared thermal switch validation study was carried out to focus on the validity of the presented computerized algorithm by isolating the algorithm-dependent errors from user-dependent errors.

#### B.2. Setup

**Figure 2:**
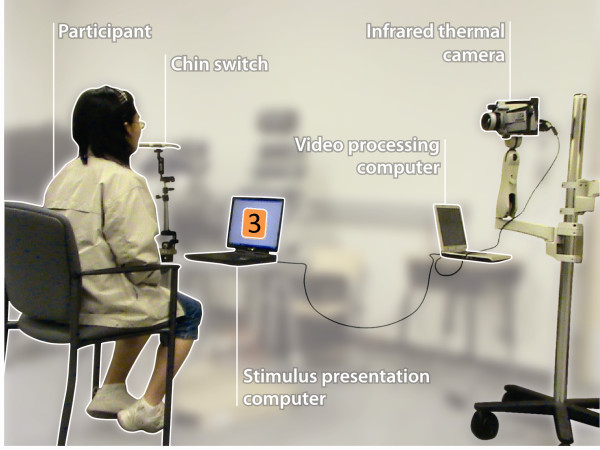
**Infrared thermal switch validity testing experiment setup**.

User performance with the infrared thermal switch was compared with a chin switch during a stimulus-response task. The chin switch was selected as the benchmark because of its guaranteed activation upon application of force and also because it can be activated by exactly the same motion that triggers the infrared thermal switch, i.e., mouth opening, therefore allowing for the study of concurrent validity of the two switches. The chin switch (Ablenet Flex Switch - product #58550) was mounted on a table in front of the user with a Slim Armstrong mounting system such that the switch sat slightly below the user's chin when his/her mouth was closed, and it came in contact with the user's chin when he/she opened his/her mouth. The infrared thermal camera (FLIR ThermaCam SC640) was positioned anterior and lateral to the participant at a 45°angle. Figure 2 depicts the experiment setup.

#### B.3. Participants

The participants were ten able-bodied university students, (23.5 ± 2.12 years old, 6 females). None had prior experience with either a chin or infrared thermal switch. The purpose of the study was explained to the participants prior to the study and they provided written consent to participate. The participants' knowledge of the purpose of the study was unlikely to bias their test performance and their views on advantages and disadvantages of switches. Since the study objective was algorithm validation, we needed to focus on algorithm-dependent errors. Hence, able-bodied participants were recruited to minimize user-dependent errors.

#### B.4. Protocol

The protocol consisted of 3 test sessions on separate days, with 6 trials per session. Each trial was 3 minutes in length. The task was to select a target number assigned by the experimenter from a pseudo-random sequence of numbers by opening and closing the mouth. Each sequence of numbers involved 60 cues out of which 10 were actionable cues, i.e., 10 target numbers for which the participant was expected to activate the switches. The numbers were presented every 2500 milliseconds, and in three stimulus modalities, namely, visual (i.e., number displayed on a computer screen), auditory (i.e., number articulated in a synthesized voice via the computer speakers), and audiovisual (i.e., both modalities simultaneously). In each session, there were two trials of each modality. The participants were asked to open and close their mouth upon observing and/or hearing the target number. The presentation modality order was randomized throughout the trials to minimize bias. To avoid learning or habituation effects, a different target number was used for each session. Overall, each participant received 180 actionable cues (3 sessions, 6 trials, 10 actionable cues). During each trial, the following data were automatically logged for both the chin and the infrared thermal switches: stimulus presentation time, response time, number of hits (true positives), true negatives, misses (false negatives), and false alarms (false positives). The protocol was approved by the Research Ethics Board of Holland-Bloorview Kids Rehabilitation Hospital and University of Toronto.

At the end of every session, the participants were asked to provide feedback about their experience with each switch. The participants were asked questions to solicit their subjective feedback and the research personnel took written notes of their answers. Specifically, they were asked to comment on their preference of switch type based on the postural requirements of switch use, the post-session level of fatigue, and the perceived disadvantages of using each switch over an extended period of time.

#### B.5. Data Analysis

A mutual information measure (MI) [21], previously reported for single switch assessment was used to objectively gauge the performance of each user. MI encapsulates different performance measures in a single number. Based on the data collected in each trial, MI was calculated for the participant's chin switch performance and separately for his/her performance with the infrared thermal switch. MI was calculated from the number of true positives, true negatives, false positives and false negatives as shown in equation (2), where *H*(*X*) is the entropy of the stimulus, *H*(*Y*) is the entropy of the switch, and *H*(*X, Y*) is their joint entropy.

(2) MI=H(X)−[H(X,Y)−H(Y)]=H(X)+H(Y)−H(X,Y)

According to [21], the maximum MI is obtained when no false negatives (misses) or false positives (false alarms) occur, in which case MI = H(X). Information transmission efficiency is thus calculated as:

(3) $$\text{Efficiency}=\frac{MI}{H(X)}$$

To rigorously assess if user performance with the infrared thermal switch was significantly different from his/her performance with the chin switch, a two-sample Kolmogorov-Smirnov (KS) test with a 5% significance level was conducted. Two-way repeated measures analyses of variance investigated the effects of switch type, stimulus modality and their interaction on efficiency, and response time.

## Results

**Figure 3:**
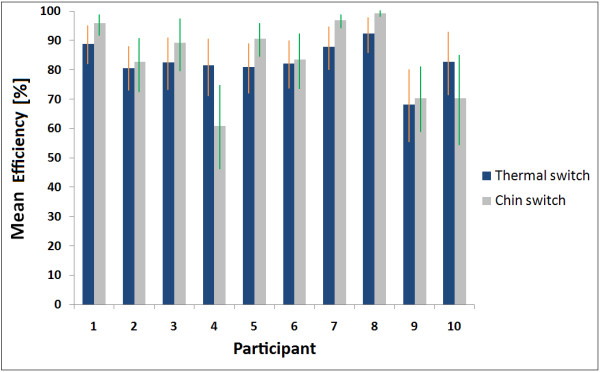
**Efficiency of infrared thermal and chin switches averaged over all eighteen trials, for all ten participants**. The vertical lines show standard deviation.

A plot of the participants' efficiency with the infrared thermal and chin switches averaged over all eighteen trials is shown in Figure 3. From visual inspection, the chin switch seems to exhibit higher efficiency than the infrared thermal switch in most cases. However, a two sample KS test indicated no significant differences between the efficiency measure for the two switches, across participants (p = 0.16).

The two-way repeated measures ANOVA overwhelmingly indicated that there were no main effects of switch type (p = 0.98) or presentation modality (p = 0.78) on efficiency and no effect of their interaction on efficiency (p = 0.998).

The two-way repeated measures ANOVA also revealed no main effects of switch type (p = 0.25), or stimulus presentation modality (0.82) on the response time. Also, no interaction effect of switch type and stimulus presentation modality on the response time was found (p = 0.997).

## Discussion

All participants achieved comparably high efficiencies with both the chin and the infrared thermal switches. From Figure 3, all participants except one achieved an information transmission efficiency of greater than 80% with the infrared thermal switch. The two-sample KS test confirmed the validity of the infrared thermal switch.

From qualitative observations of participant behavior, we noticed that during the trials, participant 4 gradually tended to lean back in his seat, such that his chin became misaligned with the chin switch. As a result, his chin did not contact the switch when he opened his mouth. This resulted in many chin switch misses and thus the chin switch efficiency dropped as shown in Figure 3. Participant 10 reported difficulty in maintaining a posture that did not activate the chin switch unintentionally. This participant only opened her mouth slightly during the task, meaning that there was very limited chin excursion. As a consequence, she leaned forward to keep her chin very close to the chin switch. This physical arrangement generated many false positives as her chin contacted the chin switch even when her mouth was closed. Unsurprisingly, chin switch efficiency declined. Remarkably, in both these instances, the infrared thermal switch maintained a greater than 80% efficiency. These examples demonstrate that the infrared thermal switch is more robust than the conventional chin switch to user motion and changes in user posture. Thus, the infrared thermal switch may be a more appropriate option for people with severe and multiple motor disabilities who may have involuntary and spastic movements.

The absence of switch type and stimulus modality effects on performance efficiency implies that the infrared thermal switch can be as useful as a conventional chin switch, regardless of the sensory modality in which the information is presented to the switch user.

Most of the participants reported that the audiovisual stimulus modality was the easiest to follow and respond to, while the audio only stimulus was the most difficult. This agrees with the recent literature contending that the response time to bi-modal stimuli (e.g., audiovisual) is shorter than the response time to uni-modal stimuli (e.g., visual or auditory alone) [22]. Some participants found the combination of chin switch and audio stimulus particularly challenging. Those participants closed their eyes and intently listened to the audio stimuli. While having their eyes closed did not affect their infrared thermal switch performance, it did cause participants to miss several chin switch activations simply because they lost track of the switch's physical location. This finding highlights the convenience of the infrared thermal switch for patients who may have vision impairments; they do not need to actively track the location of the switch relative to their chin.

Table 1 summarizes a list of switch pros and cons as indicated by the participants in their qualitative feedback. All participants preferred the flexibility of the infrared thermal switch over the chin switch. They found it difficult to maintain a stationary position at all times, as required for accurate chin switch use. Arguably, with some users with disabilities who have limited movement, such as those with muscular dystrophy, maintaining a stationary position would not be an issue. The non-contact nature of the infrared thermal switch was another major plus reported by the participants in this study.

**Table 1:** Selected qualitative feedback (pros and cons) from participants.

	Infrared Thermal Switch	Chin Switch
	Flexibility: switch works even in spite of user body motion and head rotation (as long as user face is in camera's field of view)	Instantaneous activation
	
**Pros's**	Non-contact	More sensitive switch
	
	Possible to activate the switch with closed eyes	

	Reduced detection sensitivity immediately after taking cold drink or food (this problem is alleviated with time)	Low detection sensitivity due to considerable head rotation or change of posture
	
**Con's**	Reduced detection sensitivity if too much saliva in the mouth (this problem is alleviated with one swallow)	Too many false positives due to small head motion, talking or any activity that makes the chin touch the switch
	
	Prolonged periods of switch operation may cause fatigue or jaw pain	May cause infection if it enters user mouth May cause skin irritation

There are further advantages associated with the infrared thermal switch. Being a non-contact access technology, it is hygenic and does not involve the risk of contact injury. Infrared thermography is lighting and color invariant [23]. Thus the infrared thermal switch can be used by people of all ethnicities, night or day, regardless of ambient lighting conditions. These benefits make the infrared thermal access technology an appealing access tool for people with severe motor disabilities. Any individual with voluntary control of his or her mouth movement may be a candidate for infrared thermal switch use. Indeed, like other binary switches, the infrared thermal switch can facilitate typing, game playing and communication, among other activities. Despite these benefits, there are limitations associated with this new technology, such as those indicated in Table 1. The findings in the present study are based on a modest sample of ten able-bodied adults. However, the small between-subject variation in efficiency values suggests that the sample was quite homogenous in terms of switch usage.

The practical issue of cost is worthy of mention. While fully radiometric infrared thermal imaging is considered a costly technology, the proposed infrared thermal switch can be affordably implemented given that only a grayscale intensity representation rather than the absolute temperature of the client's face is required. Hence, a less costly (approximately 2000 USD) non-radiometric camera can be used. In addition to the camera, very simple and inexpensive hardware (e.g. module latching relay and USB switch converter) have been used in the design of the infrared thermal switch. Infrared thermal cameras are usually designed to withstand harsh climates and rough handling [14]. Nonetheless, repairing an infrared thermal camera that is beyond its warranty period can be a costly undertaking.

In addition to cost, the required in situ technical adjustments should also be considered. The client's orofacial temperature is likely to vary subsequent to the consumption of food or drink or with changes in ambient temperature. As a consequence, recalibration of the client's intensity and motion thresholds may be required at the start of each session.

## Conclusions

In this paper, we presented an experiment with 10 able-bodied individuals to validate a novel infrared thermal switch algorithm. A previously proposed mutual information measure was used to quantify user performance. Users completed multiple trials of a number identification task using both infrared thermal and conventional chin switches concurrently. User performance was comparable between the proposed thermal switch and the gold standard chin switch, establishing the concurrent validity of the former. This is a clinically positive result given that the thermal switch offers the additional benefits of being non-contact and robust to user posture. The infrared thermal switch is valid with respect to a conventional chin switch and ought to be considered for clients with severe disabilities who retain voluntary mouth opening/closing.

## Competing interests

The authors declare that they have no competing interests.

## Authors' contributions

NM designed and implemented the infrared thermal switch, carried out the validation study, and drafted the manuscript. ANV read the manuscript and commented on the methods. TC conceived of the study, and participated in its design and coordination and edited the manuscript. All authors read and approved the final manuscript.
